# Correlation Between the Presence of Antinuclear Antibodies and Recurrent Pregnancy Loss: A Mini Review

**DOI:** 10.3389/fendo.2022.873286

**Published:** 2022-05-04

**Authors:** Ting Liu, Xi Guo, Ying Liao, Yingyu Liu, Yuanfang Zhu, Xiaoyan Chen

**Affiliations:** ^1^ Department of Obstetrics and Gynaecology, Shenzhen Baoan Women’s and Children’s Hospital, Jinan University, Shenzhen, China; ^2^ Department of Obstetrics and Gynaecology, The Chinese University of Hong Kong, Shatin, Hong Kong SAR, China

**Keywords:** antinuclear antibodies (ANAs), recurrent pregnancy loss (RPL), prognostic value, pregnancy outcome, immunotherapy

## Abstract

In the past decade, the incidence of recurrent pregnancy loss (RPL) has increased significantly, and immunological disorders have been considered as one of the possible causes contributing to RPL. The presence of antinuclear antibodies (ANAs) is regarded as a typical antibody of autoimmunity. However, the relationship between the presence of ANAs and RPL, the underlying mechanism, and the possible role of immunotherapy is still controversial. The aim of this mini review is to assess the association between ANAs and RPL and the effects of immunotherapy on pregnancy outcomes in women with positive ANAs and a history of RPL from the available data and to provide a relevant reference basis for clinical application in this group of women.

## Introduction

Pregnancy loss, or the spontaneous death of a pregnancy before the fetus reaches viability, affects up to 20% of women who conceive, making it one of the commonest complications of pregnancy (1). Currently, the definitions of recurrent pregnancy loss (RPL) vary in different countries and regions internationally. Based on the European Society of Human Reproduction and Embryology (ESHRE) guideline, RPL was defined as the loss of two or more pregnancies before 24 weeks of pregnancy (1), while the Royal College of Obstetricians and Gynaecologists (RCOG) guideline used a stricter criterion, which was defined as three or more fetal losses before 24 weeks of pregnancy, including biochemical pregnancy (2). Compared with pregnancy loss, RPL is less prevalent and affects approximately 1 to 3% of women who are trying to conceive (3). RPL has a significant negative impact on the physiological and psychological health of women and brings great emotional frustration to couples.

There are several recognized causes related to RPL, namely, genetic factors, uterine abnormalities (congenital malformations, endometrial polyps, uterine fibroids, etc.), hormonal and metabolic disorders (thyroid dysfunction, diabetes, polycystic ovary syndrome, hyper-prolactinemia, etc.), thrombophilia, immunological disorders, and male factors. However, approximately half of RPL remains unexplained in etiology, which is referred to as unexplained RPL (uRPL) (4).

Antinuclear antibodies (ANAs) are a group of autoantibodies that target components of the cell nucleus and bind to proteins, nucleic acids, and protein–nucleic acid complexes (5). ANA detection may be performed by immunofluorescence (IF) on human epithelial laryngeal carcinoma type 2 cells or by solid-phase ANA screening immunoassay with at least equivalent performance (6). Indirect immunofluorescence is an extensively used laboratory test for detecting ANAs. The result is usually expressed in titers, which are used to describe the antibody concentration in peripheral blood. Positive ANAs expressed in low titers are commonly found in healthy women, whereas the presence of high titers (>1:160) is closely related to autoimmune diseases, namely, systemic lupus erythematosus, systemic sclerosis, and Sjögren’s syndrome, which are related to adverse pregnancy outcomes (7, 8). A previous cross-sectional analysis including 4,754 individuals from the US showed the prevalence of positive ANAs could reach up to 13.8% and vary widely in healthy populations, ranging from 5.92% in Chinese to 30.8% in African Americans, which was higher in women (17.8%), compared with that in men (9.6%) (9).

There is evidence that autoimmunity is an important risk factor for pregnancy loss. A series of studies have tried to elucidate the association between ANAs and RPL, but the relationship between ANAs and RPL pregnancy outcomes and whether the treatments for ANA-positive affect pregnancy outcomes are still highly controversial. However, the prognostic value of ANAs for subsequent pregnancy outcomes is unclear as well. Furthermore, the underlying patho-physiological link and mechanism that the presence of ANAs plays in women with RPL has not yet been fully understood.

Therefore, given the importance of the potential association between the presence of antinuclear antibodies and pregnancy loss, the aim of this mini review was to provide evidence on the relationship between positive ANAs and recurrent pregnancy loss and the possible underlying mechanism. Given the possible role of immunotherapy in improving pregnancy outcomes in women with a history of RPL, we also reviewed the available clinical studies on the effects of different types of immunotherapy, focusing on positive ANAs.

## Presence of ANAs and its Prognostic Value in RPL

The presence of ANAs has been regarded as a typical feature of autoimmunity. There is growing evidence suggesting that ANAs can play a role in both early pregnancy and pregnancy loss. Although how the ANAs are present in women with RPL remains unclear, it is possible that the presence of ANAs in RPL indicates that there may be an underlying autoimmune disorder in RPL women, at least in a subgroup of patients, which affects the development of the trophoblast and can lead to early pregnancy loss. Therefore, RPL women with previous autoimmune diseases are likely to have a higher prevalence of positive ANAs. One previous study showed that in women with autoimmune disorders, a history of RPL is independently associated with reactivity against three distinct Ro antigen-related reactivities (a subtype of autoantibody of ANAs), suggesting that cumulative autoimmune responses correlate with the risk of spontaneous miscarriage (10). However, even in RPL women without autoimmune disorders, ANAs still need to be screened. A recent meta-analysis, including 2,683 women with RPL without defined autoimmune diseases and 2,355 controls, found that the total positive rate of ANAs was significantly higher in the RPL group, compared with the control group (22.0% vs 8.3%, OR = 2.97, 95% CI 1.91–4.64, P <0.001) (11). Additionally, subgroup analysis demonstrated a significant association between high ANA titers (≥1:160) and RPL (OR = 45.89, 95% CI 8.44–249.45, P <0.001), while there was no significant relationship between low titers of ANAs (1:40 ≤ANA ≤1:80) and RPL (OR = 2.44, 95% CI 0.42–14.06, P = 0.32).

In the other previous studies, most of them did not provide definite information on the past history of autoimmune disorders, and the results showed that the prevalence of positive ANAs in women with a history of RPL varied (**Table 1**). Several previous studies have found a significantly increased prevalence of positive ANAs in women with a history of RPL. There was a significantly higher proportion of women with RPL who had ANAs at ≥1:80 compared with controls (21, 23, 30). A case–control study including 294 women showed that women with RPL had a three-fold higher prevalence of positive ANAs (50%) and higher serum titers of ANAs (≥1:80) when compared with women without reproductive disorders (21). Similarly, another study including 560 Iranian women showed that the ANA-positive rate in women with a history of two or more unexplained pregnancy losses (13.21%) was significantly higher than that in control women (0.9%) (24). This observation was also supported by another systematic review and meta-analysis (31). Their results showed that the prevalence of positive ANAs in the RPL women (20.6%, 288/1,400) was significantly higher than it was non-pregnant women with no history of pregnancy loss (6.7%, 72/1,080) (31).

**Table 1:** The prevalence of positive ANAs in women with a history of RPL in different studies.

Author	Year	Ethnic/Country	Study subjects	Definition of RPL (the number of pregnancy loss)	ANA detection methods (cut-off dilution)	Prevalence of ANA+ (case group)	Prevalence of ANA+ (control group)
Hefler-Frischmuth et al. (12)	2017	Caucasian	Case: 114 RPL	≥3	ELISA (unclear)	NA	NA
			Control: 107 age-matched healthy controls				
Sakthiswary et al. (13)	2015	Malaysia	Case: 68 uRPL	≥2	IF (1:80)	35.3%	13.3%
			Control: 60 non-pregnant women without pregnancy				
Molazadeh et al. (14)	2014	Iran	Case: 560 uRPL	≥2	IF (1:40)	13.2%	0.9%
			Control: 560 healthy controls				
Roye-Green et al. (15)	2011	Jamaica	Case: 50 RPL	≥2	IF (unclear)	2%	2.2%
			Control: 135 multiparous women without pregnancy loss				
Ticconi et al. (16)	2010	Caucasian	Case: 194 RPL	≥2	IF (1:80)	50%	16%
			Control: 100 non-pregnant controls				
Giasuddin (17)	2010	Bangladesh	Case: 35 RPL	≥3	ELISA (unclear)	20%	0.54%
			Control: 37 normal pregnant women				
Bustos et al. (18)	2006	Argentina	Case: 118 RPL	≥3	IF (1:40)	16%	14%
			Control: 125 fertile control women without abortions and two children				
Habara et al. (19)	2002	Japan	Case: 49 uRPL	≥3	IF (unclear)	NA	NA
			Control: 72 normal women with sterility caused by male factor				
Matsubayashi et al. (20)	2001	Japan	Case: 273 RPL	≥2	IF (1:80)	23.4%	13%
			Control: 200 healthy non-pregnant women				
Kaider et al. (21)	1999	USA	Case: 302 RPL	≥3	ELISA (unclear)	45.7%	10%
			Control: 20 healthy fertile women				
Kovács et al. (22)	1999	Hungary	Case: 59 uRPL	≥2	IF (unclear)	3.39%	8%
			Control: 25 non-pregnant women without pregnancy				
Stern et al. (23)	1998	New Zealand	Case: 97 RPL	≥3	IF (1:80)	22.7%	9.4%
			Control: 106 fertile controls				
Konidaris et al. (24)	1994	Greece	Case: 44 uRPL	≥3	IF (1:40)	9.1%	2.9%
			Control: 4 non-pregnant healthy women without pregnancy loss				
Bahar et al. (25)	1993	Kuwait	Case: 103 uRPL	≥3	IF (1:40)	13.6%	1.2%
			Control: 85 multiparous non-pregnant women without pregnancy loss				
Kwak et al. (26)	1992	USA	Case: 153 uRPL	≥3	IF (1:40)	19.0%	14.0%
			Control: 90 normal controls				
Harger et al. (27)	1989	USA	Case: 277 RPL	≥2	IF (1:40)	16.3%	16.8%/16.6%
			Control: 199 non-pregnant/299 pregnant women				
Petri et al. (28)	1987	USA	Case: 44 uRPL	≥3	IF (1:40)	16%	20%
			Control: 40 Volunteers				
Garcia-De La Torre et al. (29)	1984	Mexico	Case: 20 uRPL	≥3	IF (1:20)	30%	6.6%
			Control: 30 women with normal pregnancy				

NA, Not Applicable.

Nevertheless, some other studies failed to find such a significant difference between women with RPL and controls. A case–control study including 72 Bangladeshi women showed that the mean serum levels of ANAs in women with RPL (1.07 ± 0.34) were similar in cases compared with controls (0.92 ± 0.15) (17). Another study including 243 Caucasian Argentine healthy women showed that the ANA-positive rate in women with a history of three or more unexplained pregnancy losses (16%) was similar to that in control women (14%), and the median titers (1:40) (16). A recent study including 114 women with RPL and 107 healthy controls found no significant differences were ascertained regarding serum levels of ANAs (0.32 vs 0.39, P = 0.2) (27).

It is also interesting to evaluate the association of ANAs with gene polymorphisms of the hemostasis system and RPL. Hereditary thrombophilia, namely, factor V Leiden mutation, prothrombin mutation, protein C, protein S, and antithrombin deficiency, could be associated with adverse obstetric outcomes. There was one previous study investigating the presence of autoimmune antibodies (antithyroid antibodies and ANAs) and polymorphism genotypes for factor V Leiden, prothrombin gene mutation, and MTHFR in women with RPL (14). The results showed that only one out of 39 subjects had a combination of hereditary thrombophilia and positive autoimmune antibodies, suggesting a weak association between ANAs and gene polymorphisms of the hemostasis system and RPL (14). Another study evaluating thrombophilia and immunological disorders in pregnancies found that the presence of ANAs was significantly elevated in pregnancies complicated by small for gestational age, while the prevalence of inherited thrombophilia did not differ significantly. However, the authors did not provide information on the history of miscarriage of the participants (12).

Regarding the prognostic value of ANAs, there are several studies reporting different results. An earlier study found a higher subsequent miscarriage rate in ANA-positive women with RPL as compared with ANA-negative subjects (36). In another previous prospective study, Ticconi et al. investigated the ANA status in a cohort of women with unexplained RPL before pregnancy, repeated the test during the first trimester of the subsequent pregnancy, and correlated the result with the pregnancy outcome (23). Interestingly, the authors found that subsequent miscarriages occurred in women who had ANAs positive before pregnancy and remained positive in the first trimester, whereas no miscarriages were observed in women who had ANAs positive before pregnancy but turned negative in the first trimester, which suggested that the disappearance of ANA in early pregnancy could have a favorable prognostic value in the subsequent pregnancies (23).

However, some studies did not find that the presence of ANAs could predict new pregnancy losses. One study found that the live birth rate of the next pregnancy (untreated) in the RPL patients with positive ANAs at ≥1:80 (52%) was not significantly different from that in RPL patients with negative ANAs (65.6%) (30). Likewise, Ogasawara et al. observed that the ANA-positive rate in women with RPL was 17%, and the miscarriage rate in the next pregnancy was similar to that of women who had RPL and tested negative for ANAs (32). Additionally, it was reported that the occurrence of subsequent live births was not affected by ANA levels or associated thrombophilia (25).

There are several possible causes contributing to the observed difference derived from the above studies. Firstly, different definitions of RPL were used in different studies; some studies used two or more pregnancy losses (18, 21, 24, 30, 33–35) while others used three or more pregnancy losses (13, 15–17, 19, 20, 22, 26–29) to define RPL. Secondly, the subjects recruited in the above studies were from different ethnic populations, which may have led to variability. Thirdly, these studies used different methods or assays to detect ANAs; some used IF (12, 14, 16, 19–22, 24, 26, 28–30, 36) while others used ELISA (15, 17, 27). Furthermore, different criteria were applied to define positive for ANAs; some used 1:40 (16, 19, 22, 24, 26, 28, 30, 34, 35) while others used 1:80 (14, 19, 20, 36).

## Possible Mechanisms for the Presence of ANAs in RPL

Generally, autoimmune disorders may impair all stages of pregnancy, leading to implantation failure or pregnancy loss *via* different putative mechanisms (37). It has been suggested that antiphospholipid antibody (aPL) and anti-beta(2)-glycoprotein I antibody (A-β2-GPI) can lead to placental vascular thrombosis, trophoblast dysfunction, and maternal hormone imbalance (38–40) and the presence of thyroid autoantibodies may result in dysregulation of the immune system activity at the fetal-maternal interface (41–43). Although the effects of ANAs on reproductive health are widely recognized, unlike aPL, A-β2-GPI, and thyroid autoantibodies, the exact mechanism of action of ANA in RPL is not yet clear.

**Figure 1:**
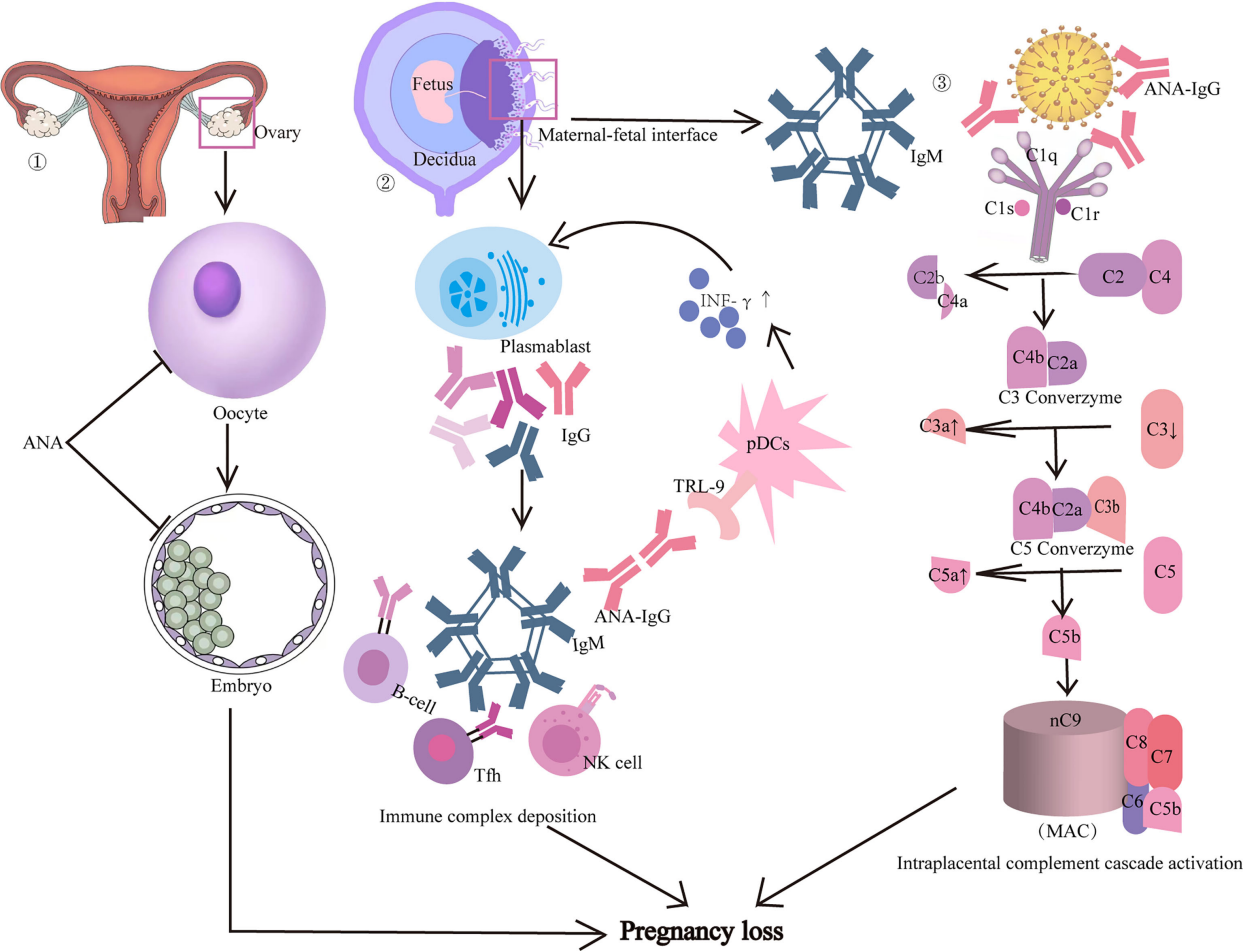
Possible mechanisms that ANA may play in pregnancy loss. Firstly, ANA might have a direct adverse effect on the quality and development of oocytes and embryos, resulting in reduced pregnancy and implantation rates. Secondly, the precipitation of immune complex tissues at the maternal–fetal interface may be one of the possible mechanisms. Thirdly, the immune complex tissues may also induce local complement activation with inflammatory infiltration, leading to miscarriage.

Previous studies have suggested several possible mechanisms that ANAs play in pregnancy failure (**Figure 1**) (44–48). Firstly, ANAs might have a direct adverse effect on the quality and development of oocytes and embryos, resulting in reduced pregnancy and implantation rates (49). Although there was no nuclear antigen on the zona pellucida, *in vitro* studies indicated that ANAs could bind to the embryos directly and it was proposed that ANAs might recognize the glycerol moiety or protein cofactor (50). An earlier study showed that the development of embryos that were co-cultured with immunoglobulins from ANA-positive women was severely impaired (51). Another study recruiting women undergoing *in vitro* fertilization-embryo transfer (IVF-ET) treatment found that the proportion of mature oocytes and number of higher embryos and pregnancy rates in the ANA-positive group was significantly lower than in the ANA-negative group, which suggested that the presence of ANAs significantly interferes with oocyte and embryo development and therefore impairs the pregnancy outcome (49).

Secondly, the precipitation of immune complex tissues in the maternal–fetal interface may be one of the possible mechanisms leading to miscarriage in ANA-positive women (51). An animal study showed that mice treated with ANA-positive IgG obtained from RPL women had a remarkably higher embryonic absorption rate, reduced complement 3 (C3) and increased C3a serum levels, compared with those treated with IgG obtained from normal healthy women (52). Interestingly, increased C3 deposition and immune complex staining in placental tissues were also found in mice treated with ANA-positive IgG fraction from women with RPL (52). Additionally, it has been shown that ANAs can also induce the activation of plasmacytoid dendritic cells *via* Toll‐like receptor‐9,which can result in increased production of inflammatory cytokines (such as interferon‐α) that stimulate the humoral immune response and lead to further production of ANAs (53, 54).

Furthermore, the immune complex tissue may induce local complement activation with inflammatory infiltration (52). Although there was no direct evidence of the association between ANAs and complement activation, a previous study using a mouse model of the antiphospholipid syndrome induced by passive transfer of human aPL antibodies showed that mice deficient in C3 were resistant to fetal injury induced by aPL antibodies (55). Studies defining the downstream effectors of complement activation have shown a rapid increase in decidual and systemic tumor necrosis factor-a levels, which appears to be the mediator that links complement activation to fetal damage (21, 56). The recruitment of inflammatory cells accelerates the activated pathway and creates a pro-inflammatory amplification loop that enhances C3 activation and deposition, generates additional C3a and C5a, and results in a further flow of inflammatory cells into the placenta, ultimately leading to pregnancy loss (56).

## Potential Treatments for ANA-Positive Women With RPL

Interventions for ANA-positive women with a history of RPL were recommended on the basis of the possible adverse effect of ANAs on the subsequent pregnancy outcome in this group of women. However, there is no consensus on treatment regimens yet.

Aspirin has both anti-platelet and anti-inflammatory effects, and glucocorticoids exhibit a beneficial clinical effect in most autoimmune diseases. Therefore, they are considered potential therapies in ANA-positive women with RPL, which has a suspected immune etiology. In a placebo-controlled trial, prednisolone and low-dose aspirin were used to treat women with RPL and positivity for antiphospholipid, antinuclear, anti-DNA, or anti-lymphocyte antibodies (57). Although the live birth rate was higher in the treatment group, it was not significantly different from controls (OR 1.5; 95% CI 0.8–2.6). However, the treated patients had a significantly higher risk of preterm birth (62% versus 12%, p <0.001) and higher risks for diabetes and hypertension, which are well known to be associated with high and prolonged administration of prednisolone. However, another case–control study, including more than 200 women who were diagnosed with unexplained RPL and tested positive for ANAs at a titer of 1:80 or more, showed that live birth rates were comparable in women receiving prednisone plus aspirin and women prescribed aspirin only. Additionally, no previous preterm delivery, fetal growth restriction, or placental abruption occurred in any subject (58).

In addition to glucocorticoids, there are other types of immunotherapy used to treat RPL with immunological causes, namely, intravenous immunoglobulin (IVIG), lymphocyte immunization treatment (LIT), etc. IVIG is a fractionated blood product that is used to treat certain autoimmune diseases and RPL. Two randomized controlled trials indicated that IVIG increases live birth rates in secondary RPL patients but not significantly in patients with primary RPL (59, 60). In contrast, recent meta-analyses found that IVIG did not improve the live birth rates of RPL women (61, 62). As for RPL women with positive ANAs, there is a limited study evaluating the therapeutic effect of IVIG in this group of women. One earlier study showed that low-dose IVIG therapy is beneficial for older women with immunologic abnormalities and RPL by increasing the successful pregnancy rate, in which 28% of the participants were found to have positive ANAs (63). The possible mechanisms of action of IVIG for treating RPL are multifactorial, namely, the modulation of various immune cells, the down-regulation of primary antibody production, and the modulation of complement activation (64–66).

LIT is another immunotherapy used in RPL. A review demonstrated that RPL women treated with paternal LIT had more successful outcomes (68%) than untreated women (54%, p <0.02) (67). As for those with positive ANAs, a retrospective observational study showed that the presence of ANAs is one of the risk factors for further pregnancy loss in patients with RPL treated with LIT (68). However, some previous studies have shown that patients with positive ANAs and antithyroid antibodies after receiving LIT have a higher risk of miscarriage and do not benefit from LIT (69, 70). Although the exact mechanisms of LIT have yet to be elucidated, the possible mechanisms consist of inducing the production of humoral antibodies to mask the fetal human leukocyte antigens (71), regulating Th2 cell transition (72), and decreasing NK cell activity (73).

Plasmapheresis has been used for decades for treating autoimmune disease as it is thought to have a profound modulation of the immune system, namely, the removal of circulating immune complexes, immunoglobulins, and complement components. Plasmapheresis has also been used in pregnant women with autoimmune diseases, such as Sjoegren’s Syndrome or systemic lupus erythematosus, to treat congenital fetal heart block (74, 75). Several reports have suggested that plasmapheresis may also treat pregnant women with anti-phospholipid syndrome (76, 77). For women with RPL, plasmapheresis was used to prevent future miscarriage in pregnant women immunized with anti-P or anti-PP1Pk and a history of RPL (78, 79). However, as far as we are aware, there is no study reporting the use of plasmapheresis in RPL women with positive ANAs.

Heparin is effective for its anticoagulant and anti-inflammatory properties (80). Due to the evidence from randomized controlled trials that heparin appears beneficial in treating women with RPL and other autoimmune antibodies (81, 82), heparin has been increasingly administered in clinic to RPL women with positive ANAs. However, there is a limited clinical trial investigating the therapeutic effect of heparin alone in this group of women. Overall, different results showed that the effect of different therapies on maternal and fetal pregnancy outcomes in patients with RPL is still controversial, and therefore large sample size randomized controlled trials are needed.

## Future Direction

As discussed above, several issues should be taken into account in future studies. Firstly, due to the different definitions used for recurrent pregnancy loss among these studies, standardization of the definition is in urgent need. Secondly, since the methods for ANA detection varied as well, a standardized methodology should be proposed in the future. Thirdly, clinical pregnancy outcomes are assumed to be followed up, namely, clinical pregnancy, miscarriage, pregnancy complications (gestational hypertension, intrauterine fetal restriction, etc.), and live birth in future studies focusing on the correlation between the presence of positive ANAs and pregnancy outcomes in women with RPL. Moreover, although a series of studies have suggested the possible roles that ANAs play in pregnancy loss, the exact mechanisms are still unclear and further mechanistic investigations are needed. *In vitro* co-culture models of endometrial and trophoblast cells may provide more information in this regard.

## Conclusion

Recurrent pregnancy loss is a challenging disease in the field of reproductive medicine that can cause great emotional frustration to the suffering couples. Although the exact mechanism that ANAs plays in women with RPL is still unclear, most studies suggest that the presence of ANAs not only correlates significantly with RPL but also has a prognostic value for the subsequent pregnancy outcome in this group of women. Interventions for ANA-positive women with a history of RPL include aspirin, glucocorticoids, and heparin. However the therapeutic effect of these regimens is still controversial and large-scale randomized controlled trials are needed.

## Author Contributions

TL, YZ, and XC: Substantial contribution to the conception and design of the work. TL, XG, YLia, and XC: participation in acquisition of the literature. TL, YLiu, YZ, and XC: manuscript drafting. All authors listed have made a substantial, direct, and intellectual contribution to the work and approved it for publication.

## Funding

This study was supported by Shenzhen Key Medical Discipline Construction Fund (SZXK028), Shenzhen Science and Technology Program (JCYJ20210324141403009) and Basic and Applied Basic Research Foundation of Guangdong Province of China (2020A1515110082).

## Conflict of Interest

The authors declare that the research was conducted in the absence of any commercial or financial relationships that could be construed as a potential conflict of interest.

## Publisher’s Note

All claims expressed in this article are solely those of the authors and do not necessarily represent those of their affiliated organizations, or those of the publisher, the editors and the reviewers. Any product that may be evaluated in this article, or claim that may be made by its manufacturer, is not guaranteed or endorsed by the publisher.
